# Intravenous Transplantation of Human Hair Follicle-Derived Mesenchymal Stem Cells Ameliorates Trabecular Bone Loss in Osteoporotic Mice

**DOI:** 10.3389/fcell.2022.814949

**Published:** 2022-03-10

**Authors:** Longshuai Lin, Enjun He, Hongjie Wang, Weihong Guo, Zhenkai Wu, Kai Huang, Qinghua Zhao

**Affiliations:** ^1^ Department of Orthopedics, Shanghai General Hospital, Shanghai Jiao Tong University School of Medicine, Shanghai, China; ^2^ Department of Pediatric Orthopaedics, Shanghai Xinhua Hospital, Shanghai Jiao Tong University School of Medicine, Shanghai, China; ^3^ Department of Orthopedics, Zhabei Central Hospital of Jing’an District, Shanghai, China

**Keywords:** hair follicle, mesenchymal stem cells, osteoporosis, bone remodeling, OPG, RANKL, noggin

## Abstract

**Background:** Hair follicles harbor a rich autologous stem cell pool and human hair follicle-derived mesenchymal stem cells (hHF-MSCs) have multi-lineage differentiation potential. Many sources of MSCs include hHF-MSCs have been attractive candidates for cell therapy, regenerative medicine and tissue engineering. The present study is to explore the effect of intravenous transplantation of hHF-MSCs on bone mass in osteoporotic mice and its mechanism, and provides prospects for clinical applications for the treatment of osteoporosis with hHF-MSCs.

**Methods:** Physically pull out about 20 hairs with intact hair follicles from the occipital area of the scalp of healthy volunteers, and extract hair follicle-derived fibroblast-like cells. These cells were cultured and characterized *in vitro*. Intravenous injection of hHF-MSCs was performed on ovariectomy-induced and age-related osteoporotic SCID mice for osteoporosis treatment. The mice were sacrificed 7 weeks after the second injection and samples were collected. The long bones and L1 vertebrae were collected for micro-CT scan, histomorphometry and immunohistochemical analysis. Peripheral serum were collected for ELISA analysis and antibody array.

**Results:** Hair follicle-derived fibroblast-like cells were defined as hHF-MSCs. Intravenous transplantation of hHF-MSCs can better restores trabecular bone mass in osteoporotic mice. The double calcein labeling assay, trap staining of bones and ELISA analysis in peripheral serum showed enhanced bone formation and weakened bone resorption after transplantation. Antibody array and immunohistochemical analysis showed that several cytokines including OPG, Wnt2b, Noggin, VCAM-1 and RANKL might be involved in this process.

**Conclusion:** Human HF-MSCs transplantation can combat trabecular bone loss induced by menopause and aging in mice. And the above mechanism that hHF-MSCs transplantation inhibits bone resorption and promote bone formation is related to OPG, Wnt2b, VCAM-1, Noggin and RANKL.

## Introduction

Mesenchymal stem cells (MSCs) are multipotent stem cells which are capable to self-renew and differentiate into different kinds of tissues (Caplan, 2007; da Silva Meirelles et al., 2006), including bone, muscle, adipose, tendons, neurons, and myocardium, under specific *in vivo* and *in vitro* conditions (Bruder et al., 1998; Ferrari et al., 1998; Young et al., 1998; Dennis et al., 1999; Qian and Saltzman, 2004; Miao et al., 2017). Compared with other stem cell types, MSCs possess great advantages since they are relatively less low immunogenic to recipients and have a powerful immunosuppressive secretion (Peng et al., 2013). MSCs modulate bone remodeling, enhance angiogenesis, reduce inflammation and promote tissue repair through autocrine/paracrine pattern. In addition, MSCs have no risk of teratoma formation and no ethical issues (Kassem et al., 2004; Augello et al., 2010). All of these properties make MSCs an attractive candidate for cell therapy, regenerative medicine and tissue engineering.

Currently, many sources of MSCs have been used in the clinical setting. They are generally divided into: adult MSCs from bone marrow (BM), adipose tissue, peripheral blood, and dental pulp, and the neonatal tissue-derived MSCs from placenta, amnion, and umbilical cord (Rodríguez-Fuentes et al., 2021). Bone marrow-derived mesenchymal stem cells (BM-MSCs) are the most widely investigated, which are believed to contribute to cartilage and subchondral bone repair in knee osteoarthritis animal models. Similar preclinical animal studies were reported for rheumatic, cardiovascular, respiratory, and metabolic diseases (Matthay et al., 2010; Kim et al., 2015; Tyndall, 2015; José et al., 2017). Adipose tissue-derived mesenchymal stem cells (AT-MSCs) may be useful for cell-based therapy for radiculopathy and neuropathic pain (Yang et al., 2010; Berebichez-Fridman et al., 2017; Majka et al., 2017). Hair follicles are easily accessible and harbor a rich autologous stem cell pool with multi-lineage (myogenic, osteogenic, adipogenic and chondrogenic lineages) differentiation potential. Compared with BM-MSCs, HF-MSCs have a higher proliferative capacity (Bajpai et al., 2012) and have been used as cell sources to engineer functional vascular grafts (Peng et al., 2011), to re-establish hematopoietic tissues (Lako et al., 2002), to reconstruct full-thickness skin (Michalak-Micka et al., 2019) and to deliver release-controlled insulin gene (Wu et al., 2015). More promising results of hair follicles have been obtained from both clinical and preclinical settings.

In the last few decades, the mean life expectancy has increased which consequently boosted the impact of skeletal diseases. Osteoporosis is the most common disease involving bone degeneration and has placed a tremendous burden on the health care system (Reginster and Burlet, 2006; Harvey et al., 2010). There are two subtypes of primary osteoporosis, type I and type II, which are also known as postmenopausal and senile osteoporosis (Wang et al., 2012). Indeed, various treatment strategies, such as anti-resorptive drugs, anabolic drugs and other antibody therapies, have been developed to reduce bone loss and prevent fractures (Rachner et al., 2011). Despite these remarkable advances, its side-effects limits the use of drugs (Khan et al., 2017; Khosla and Hofbauer, 2017; Camacho et al., 2020), suggesting that discovering new targets for the prevention and treatment of osteoporosis is of importance and it is plausible to consider strategies targeting both bone formation and resorption for efficient and long-term protection.

MSCs are promising cells for cell therapy in bone diseases because of their features such as self-renewal and plasticity. Also, unlike embryonic stem cells, the use of MSCs does not raise any ethical issues. Studies have shown BM-MSCs could improve bone formation and stimulate osteoblastogenesis (Sui et al., 2016). AT-MSCs could also increase trabecular number and raise bone marrow density (BMD) (You et al., 2012). However, the contribution of HF-MSCs to bone homeostasis and osteoporosis is unknown.

Here, we made attempts to examine whether hHF-MSCs transplantation via systemic infusion could ameliorate the ovariectomy (OVX) and aging-induced bone loss in mouse models. And the distribution of exogenous hHF-MSCs in heart, lung, liver, kidney and bone of mice was also studied. Moreover, the molecular mechanisms underlying the effects of exogenous hHF-MSCs on bone mass of mice were investigated preliminarily through the evaluation of bone remodeling status and expression of bone metabolism regulators in serum and bone marrow in OVX-induced osteoporotic mice in response to intravenous transplantation of hHF-MSCs. We uncovered a novel therapeutic potential of human HF-MSCs transplantation in osteoporosis. Our study will benefit not only for deep insights into the biological features and medical applications of HF-MSCs, but also for providing new therapy targets to treat osteoporosis.

## Materials and Methods

### Isolation and Culture of hHF-MSCs

All protocols of human tissue handling were approved by the Ethics Committee of Shanghai General Hospital, Shanghai Jiaotong University. Four healthy volunteers (two females and two males in 25–35 age) were recruited for every round of experiments. At least 20 hairs with complete hair follicles were physically plucked from the occipital region of the scalps. Hairs were intensively rinsed with phosphate buffered saline (PBS) containing 1% penicillin/streptomycin solution (Gibco BRL, Rockville, MD, United States) three times. Then hair shafts were cut off and hair follicles were transferred onto the bottom of a 24-well plate (Corning, Tewksbury, MA, United States), one follicle per well, and maintained in DMEM/F-12 (Life Technologies, Madison, Wi, United States) supplemented with 10% fetal bovine serum (FBS; Hyclone, Victoria, Australia), 10 ng/ml basic fibroblast growth factor (bFGF; PeproTech, London, United Kingdom) in a 37°C/5% CO_2_ incubator. The medium was replaced every 3 days. Five to ten days later, fibroblast-like cells migrated out of the dermal sheath or papilla. Passage 4–6 cells were used in the following experiments.

### Flow Cytometry

The cells were detached from the culture flasks with trypsin-EDTA and counted. About 1 × 10^6^ cells were incubated on ice for 30 min with goat serum. Subsequently, the cells were stained for 30 min at 4°C with specific antibodies. Isotype-matched antibodies (BD-pharmingen, United States) were used to rule out non-specific staining of the cells. The labeled cells were thoroughly washed with PBS and analyzed on a BD Calibur machine (BD Biosciences, United States) using the Cell quest as data acquisition software.

### Osteogenic, Adipogenic, and Chondrogenic Differentiation of hHF-MSCs

For osteogenic differentiation, BMSCs were treated with 100 nM dexamethasone (Dex), 10 mM β-glycerophosphate disodium and 50 μg/ml ascorbic acid for 4 weeks. After induction, alkaline phosphatase (ALP) and alizarin red (AR) staining were performed (Zhao et al., 2013). For adipogenic differentiation, confluent BMSCs were fed with complete adipogenic hormone cocktail, i.e., α-MEM supplemented with 10% FBS, 10 g/ml of insulin, 0.5 mM methylisobutylxanthine (MIX) and 1 μM Dex (All from Sigma). The start point of differentiation was referred to as day 0. On day 3, cells were fed with α-MEM containing only insulin and 10% FBS. On day 6, complete adipogenic hormone cocktail was again added. The whole adipogenesis induction process lasts 30 days. After induction, Oil red O staining was performed to assess fat droplets formation (Zhao et al., 2013). For chondrogenic differentiation, 20 μl of a suspension of 8 × 10^6^ hHF-MSCs/ml were allowed to form a sphere by hanging drop culture and spheres were cultured in HG-DMEM, 10% FBS, 6.25 μg/ml insulin, 10 ng/ml TGF-β1 (PeproTech, London, United Kingdom), and 50 nM of ascorbate-2-phosphate (Sigma-Aldrich) for 3 weeks. After induction, spheres were fixed in 10% buffered formaldehyde, embedded in paraffin, sectioned at 5 μm, and stained with Alcian blue (Sigma-Aldrich) (Bai et al., 2017).

### RT-qPCR

Total RNA was isolated using RNeasy Mini Kit (Qiagen, Valencia, CA, United States). For reverse transcription, single-stranded cDNA was reverse transcribed from 1 μg total RNA using oligo-dT primer. Quantitative PCR analysis was performed on a 96-well plate ABI Prism 7500 Sequence Detection system (Applied BioSystems, Foster City, CA, United States) using SYBR Green PCR Master Mix (Takara Bio Inc., Otsu, Japan). Cycling conditions was as follows 94°C, 5 s; 60°C, 34 s; and 72°C, 40 s for 40 cycles. GAPDH (osteogenesis and chondrogenesis) and HPRT (adipogenesis) were used as internal controls for RT-qPCR. Primer sequences were listed in Supplementary Table S1.

### Anti-Proliferative Properties of hHF-MSCs

Peripheral blood sample was collected from a healthy donor and PBMCs were prepared by density gradient centrifugation using Ficoll-hypaque media and were labeled with 5 nM CFSE dye (Invitrogen Molecular probe, United States). Then cells were incubated at 37°C for 10 min and centrifuged at 1,800 rpm for 10 min. Next CFSE-labeled PBMCs were counted and added into wells at a concentration of 1 × 10^6^ cells per well. 2 × 10^5^ hHF-MSCs were plated in culture plates 24 h prior to co-culture with PBMCs. PBMCs were stimulated with PMA (Gibco, United Kingdom) and the effect of hHF-MSCs on lymphocyte proliferation was studied in Tanswell co-culture system (0.4 μm Transwell plates, corning, United States). After 3 days of co-culture, PBMCs were collected and subjected to flow cytometry analysis.

### hHF-MSCs/CD4^+^ T Cells Co-Cultures

Human peripheral blood CD4^+^ T cells (Lonza, Walkersville, MD) were cultured with X-VIVO medium (Lonza) containing 2% FBS. Th1 cells were differentiated with anti-CD3-coated plates (5 μg/ml, BD Biosciences), anti-CD28 antibodies (2 μg/ml, BD Biosciences) plus IL-12 (10 ng/ml, R&D Systems, United States) and anti-IL-4 neutralizing antibodies (2.5 μg/ml, BD Biosciences) for 6 days. Th17 cells were differentiated with anti-CD3-coated plates (5 μg/ml) plus anti-CD28 (2 μg/ml), in the presence of IL-6 (50 ng/ml, R&D Systems), TGF-β1 (5 ng/ml, BioVision, United States) and anti-IFN-γ (2.5 μg/ml, BD Biosciences) and anti-IL-4 (2.5 μg/ml, BD Biosciences) neutralizing antibodies for 6 days. Treg cells were differentiated with anti-CD3-coated plates (5 μg/ml) and anti-CD28 (2 μg/ml), in presence of TGF-β1 (10 ng/ml, BioVision, United States) and anti-IFN-γ (2.5 μg/ml, BD Biosciences) and anti-IL-4 (2.5 μg/ml, BD Biosciences) neutralizing antibodies for 6 days. The effect of hHF-MSCs on Th1, Th17 and Treg differentiation was tested by co-cultivating cells at a ratio of 10:1 (T cells/hHF-MSCs) after 3 days of Th1, Th17 or Treg polarizing conditions (day 3). After 3-day co-culture of T cells and hHF-MSCs, flow cytometry was performed in order to detect IFN-γ+CD4^+^ Th1 cells, IL-17 + CD4^+^ Th17 cells, and CD4^+^CD25 + Foxp3+ Treg cells.

### Animal Models and hHF-MSCs Injection

Severe combined immunodeficient (SCID) mice were purchased from SIPPR-BK Laboratory Animal Co., Ltd. (Shanghai, China). All mice were bred and maintained under specific pathogen-free conditions in the animal facility of Shanghai General Hospital, Shanghai Jiaotong University. All experiments were performed with the protocol approved by the Animal Care and Use Committee of Shanghai General Hospital, Shanghai Jiaotong University. For the establishment of estrogen-deficiency osteoporotic mouse model, ovariectomy (OVX) was performed in SCID mice. Under general anesthesia, a scalpel was used to create a 1-cm incision on each side of the spine to expose the ovaries, which were then ligated with an absorbable suture, after which the ovariectomy surgery was performed. Sham-operated mice were used as the control group. Eight weeks after surgery, osteoporosis was developed and mice were subjected to intravenous hHF-MSCs injection. For the establishment of mouse model of age-related bone loss, 5- and 10-month-old SCID mice were used to mimic age-related bone loss, since the peak bone mass of mice is reached between 5 and 6 month of age (Weinstein et al., 1998). Mice with OVX-induced osteoporosis were divided into four groups: Sham, OVX, OVX + hHF-MSCs (Low dose) and OVX + hHF-MSCs (High dose). Mice in Sham and OVX groups received saline injection. Mice in OVX + hHF-MSCs (L) and OVX + hHF-MSCs (H) received the injection of 3 × 10^5^ and 6 × 10^5^ hHF-MSCs. Mice with age-related bone loss were divided into four groups: 5 m, 10 m, 10 m + hHF-MSCs (Low dose) and 10 m + hHF-MSCs (High dose). Mice in 5 m and 10 m groups received saline injection. Mice in 10 m + hHF-MSCs (L) and 10 m + hHF-MSCs (H) received the injection of 3 × 10^5^ and 6 × 10^5^ hHF-MSCs. On study day 0, hHF-MSCs were injected into the tail veins of mice. On day 7, second dose of hHF-MSCs were injected. Mice were sacrificed and samples were collected at week 7 after the second injection. Heart, lung, liver, and kidney were collected for *in vivo* distribution of exogenous hHF-MSCs in mice. Tibia and L1 vertebrae were collected for micro computed tomography (Micro-CT). Femurs were prepared for micro-CT scan, histomorphometry, and immunohistochemistry analysis. Peripheral blood serum was taken for antibody array analysis and the measurement of Tartrate resistant acid phosphatase-5b (Trap-5b) and propeptide of type I procollagen (P1NP) (Immunodiagnostic Systems plc, Tyne and Wear, United Kingdom).

### Micro-CT

Bone densities of femur, tibia and L1 vertebrae were measured by micro-CT (μCT-80, Scanco Medical AG, Bassersdorf, Switzerland). Standard nomenclature and guidelines for bone microstructure were employed (Bouxsein et al., 2010). The bones were scanned at an energy level of 55 kVp, intensity of 145 μA, and a fixed threshold of 220. Trabecular and cortical regions of femur were analyzed at 0.35 and 4.25 mm from the growth plate of distal femur, respectively. For both regions, 1.5 mm of femur bone sections were individually analyzed at 6 μm resolution. Trabecular regions of proximal tibia and the whole L1 vertebrae were analyzed. Three-dimensional images were reconstructed. The main parameters of trabecular bone are BV/TV (bone volume/total volume), Tb.N (trabecular bone number), and Tb.Sp (trabecular bone space). The main parameters of cortical bone are Ct.ar/Tt.ar (cortical bone area/total area).

### Histomorphometric Analysis

Mice were subcutaneously injected with calcein (Sigma, St. Louis, MO, United States) at a dose of 15 mg/kg at day 10 and day 3 before sacrifice. After fixation and embedding, femurs were cut into 50 μm thick sections and the double calcein labeling was imaged with a fluorescence microscope. BFR (bone formation rate) was calculated as inter-label width/labeling period with Image Pro software.

Bone histomorphometric analysis to quantify osteoclasts was performed in mouse femurs embedded in paraffin that were stained for Trap. Osteoclasts were identified as multinucleated Trap-positive cells adjacent to bone surface. All analysis was confined to the secondary spongiosa and restricted to an area between 500 and 2000 μm proximal to the growth plate-metaphyseal junction of the distal femur. The percentage of osteoclast surface (Oc.S) to bone surface (BS) was calculated.

### Detection of Human Alu Sequences by Genome DNA qPCR

DNA extraction was performed using the DNeasy Blood & Tissue kit (Qiagen, Courtaboeuf) and quantified using a spectrophotometer (Nanodrop, Labtech, Palaiseau). qPCR was performed on 25 ng DNA in a total volume of 10 μl that contained 5 μl of DNA Master SYBR Green I kit (Roche Diagnostics, Meylan) and 0.05 μM primers for Alu sequence or 0.5 μM for mouse β-actin. The primer sequences for human Alu sequence and mouse β-actin sequences were listed: Alu: 5′-CAT​GGT​GAA​ACC​CCG​TCT​CTA-3' (F), 5′-GCC​TCA​GCC​TCC​CGA​GTA​G-3′ (R); β-actin: 5′-CCA​CCA​TGT​ACC​CAG​GCA​TT-3′ (F), 5′-AGG​GTG​TAA​AAC​GCA​GCT​CA-3′ (R). PCR conditions were as follow: 95°C for 15 min followed by 40 cycles at 95°C for 15 s and 64°C for 30 s and then 40°C for 30 s. Standard curves were generated by adding ten-fold serial dilutions of hHF-MSCs DNA in the DNA of mouse cells (the total DNA amount was kept constant to 25 ng). The results were expressed as the percentage of hHF-MSCs DNA in mouse DNA.

### Detection of Human Alu Sequences by *in situ* Hybridization

Five-micrometer sections of decalcified femur were deparaffinized and rehydrated. After antigen retrieval, slices were washed in distilled water for 10 min at room temperature and immersed in increasing graded ethanol for 1 min. DNA was denatured at 95°C for 5 min, and hybridization process was performed with human Alu-DNA Probe (Biogenex, Fremont, CA, United States) at 37°C overnight. Hybridized slices were washed in standard saline citrate (pH 7.0; Dako) at room temperature for 10 min, 56°C for 10 min, and room temperature for 15 min. Nonspecific binding was prevented by incubation in PBS containing 3% bovine serum albumin (Sigma) for 1 min followed by a two-step avidin and biotin blocking (Vector Laboratories Ltd., Brussels, Belgium) for 15 min at room temperature. To detect hybridized Alu sequences, slices were incubated with biotinylated anti-fluorescent antibody (1/100) (Vector Laboratories Ltd.) for 1 h, and the signal was revealed using streptavidin/horseradish peroxidase (Dako) with diaminobenzidine as the chromogenic substrate.

### Antibody Array

Absolute quantitative sandwich-based antibody array (RayBio^®^) was used to detect ten bone metabolisms regulators in mice serum. The corresponding detection antibodies were biotin-labeled and combined as a single cocktail reagent for later use. After blocking arrays with a blocking buffer, they were incubated with peripheral blood serum. Following extensive washing to remove non-specific binding, the cocktail of biotinylated detection antibodies was added to the arrays. After extensive washing, the array slides were incubated with a streptavidin-conjugated fluor (HiLyte Fluor™ 532, from Anaspec, Fremont, CA, United States). Fluorescent signals was visualized using a laser-based scanner system (GenePix 4200A, Molecular Dynamics, Sunnyvale, CA, United States).

### Immunohistochemistry Analysis

Femurs were fixed with 4% PFA, decalcified with EDTA, embedded in paraffin, and cut into 3 μm sections. After treatment with Protein Block (Dako Cytomation, Glostrup, Denmark), sections were incubated with anti-mouse OPG (Thermo Fisher Scientific, Ann Arbor, MI, United States), anti-mouse VCAM-1 (Cell Signaling Technology, Danvers, MA, United States), anti-mouse RANKL (Thermo Fisher Scientific), anti-mouse Noggin (Thermo Fisher Scientific), and anti-mouse Wnt2b (Thermo Fisher Scientific) overnight at 4°C. Then slices were incubated with biotinylated anti-fluorescent antibody (Vector Laboratories Ltd.) for 1 h, and the signal was revealed using streptavidin/horseradish peroxidase (Dako) with diaminobenzidine as the chromogenic substrate and counterstained with hematoxylin.

### Statistical Analysis

Student’s t-test was used for two-sample comparisons. One- and two-way ANOVA was used for multiple comparisons. Tukey’s test was used to find significant differences in ANOVA. *p* < 0.05 was defined as significant. All data are presented as means ± s.d unless otherwise specified.

## Results

### *In vitro* Culture and Characterization of hHF-MSCs

**FIGURE 1:**
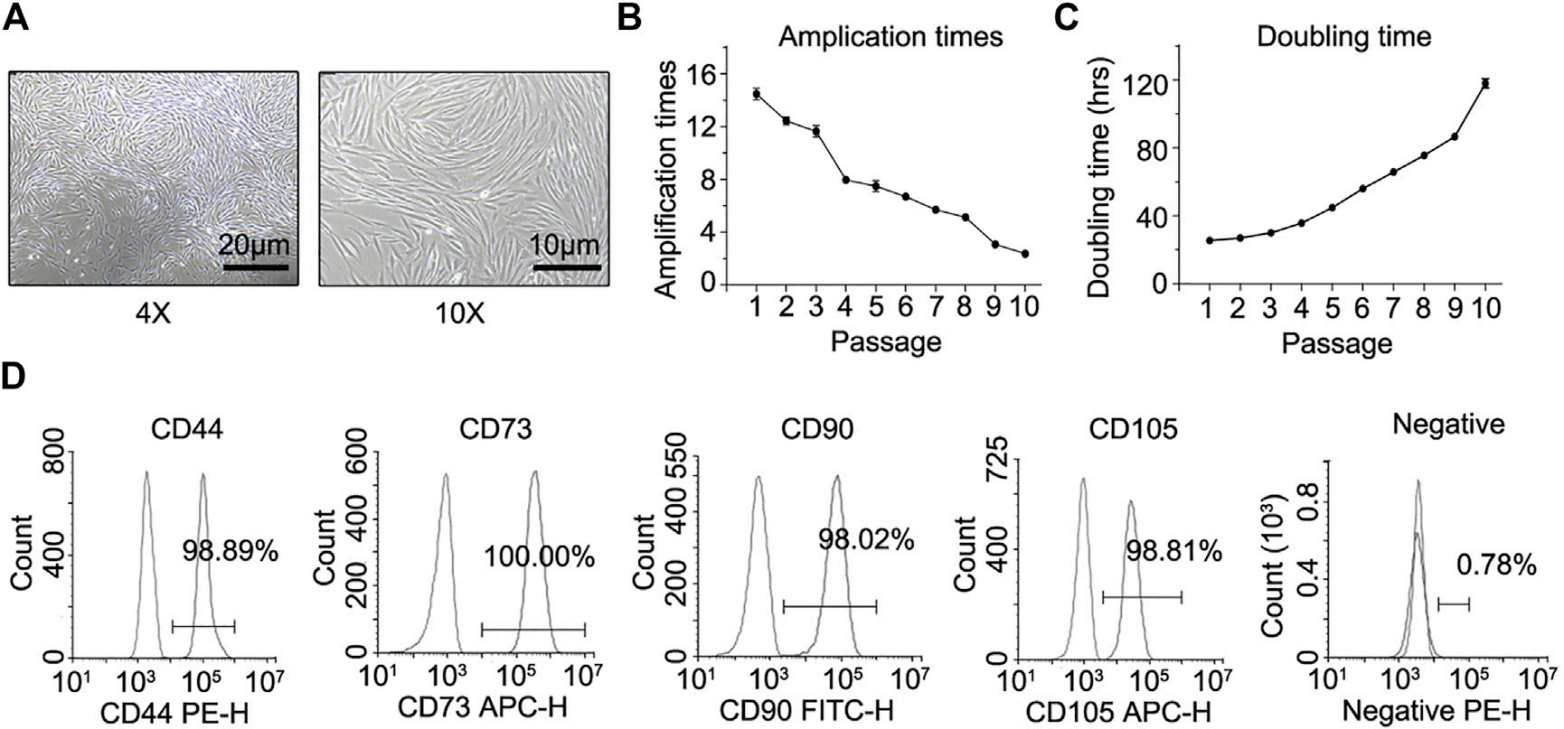
*In vitro* culture, proliferation properties, and mesenchymal lineage surface markers of hHF-MSCs. **(A)** hHF-MSCs morphology under light microscope. **(B)** Amplification times of hHF-MSCs from passage 1 to passage 10. **(C)** Doubling time of hHF-MSCs from passage 1 to passage 10. hrs: hours. **(D)** Flow cytometry analysis on mesenchymal lineage surface markers of hHF-MSCs including CD44^+^, CD73^+^, CD90^+^, CD105^+^, CD11b^−^, CD19^−^, CD34^−^, CD45^−^, and HLA-DR^-^. All the data were obtained from three independent experiments. Data were shown as the means ± s.e.m.

The isolated hair follicles adhered to the culture plate and cells migrated out of the hair follicle dermal sheath or papilla in 7–10 days, exhibiting typical fibroblast-like shape in morphology (Figure 1A). From passage 1 to 10, the amplification times of these cells declined steadily (Figure 1B). In contrast, there was a steady increase in the doubling time of these cells from passage 1 to 10 (Figure 1C). Immunofluorescence staining and flow cytometry assays showed that these fibroblast-like cells expressed surface markers of MSCs. They were positive for CD44, CD73, CD90, and CD105, but negative for CD11b, CD19, CD34, CD45, and HLA-DR (Figure 1D).

**FIGURE 2:**
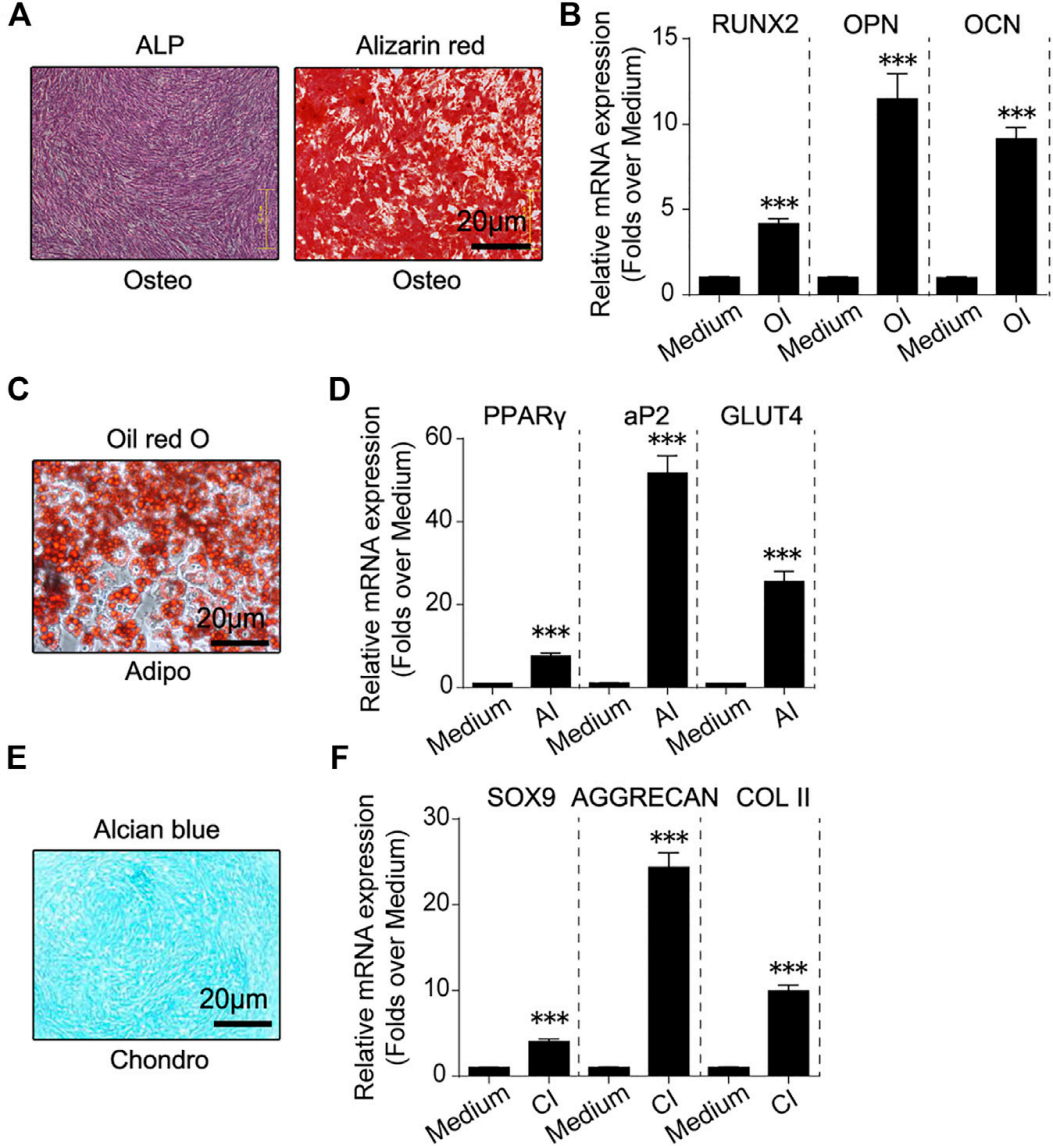
*In vitro* osteogenic, adipogenic and chondrogenic differentiation of hHF-MSCs. **(A)** ALP and Alizarin red staining images of *in vitro* hHF-MSCs cultures when induced to undergo osteogenic differentiation. **(B)** mRNA expression of osteogenesis marker genes including Runx2, Opn and Ocn in *in vitro* hHF-MSCs cultures when induced to undergo osteogenic differentiation. OI: osteogenic induction. **(C)** Oil red O staining images of *in vitro* hHF-MSCs cultures when induced to undergo adipogenic differentiation. **(D)** mRNA expression of adipogenesis marker genes including PPARγ, aP2 and Glut4 in *in vitro* hHF-MSCs cultures when induced to undergo adipogenic differentiation. AI: adipogenic induction. **(E)** Alcian blue staining images of *in vitro* hHF-MSCs cultures when induced to undergo chondrogenic differentiation. **(F)** mRNA expression of chondrogenesis marker genes including Sox9, Aggrecan and Col II in *in vitro* hHF-MSCs cultures when induced to undergo chondrogenic differentiation. CI: chondrogenic induction. GAPDH was used as internal control in RT-qPCR for osteogenesis and chondrogenesis. HPRT was used as internal control in RT-qPCR for adipogenesis. All the data were obtained from three independent experiments. Data were shown as the means ± s.e.m. *: *p* < 0.05, **: *p* < 0.01, ***: *p* < 0.001, NS: not significant.

These fibroblast-like cells were induced to undergo osteogenic, adipogenic and chondrogenic differentiation *in vitro*. Alkaline phosphatase (ALP) and alizarin red (AR) staining revealed ALP activation and calcium deposition of these fibroblast-like cells in response to osteogenic induction (Figure 2A). RT-qPCR showed that osteogenesis marker genes including Runx2, Opn and Ocn were activated in response to osteogenic induction (Figure 2B). Oil red O staining revealed fat droplets formation of these fibroblast-like cells in response to adipogenic induction and adipogenesis marker genes including PPARγ, aP2 and Glut4 were also activated (Figures 2C,D). Alcian blue staining revealed proteoglycan synthesis and secretion by these fibroblast-like cells in response to chondrogenic induction (Figure 2E). And chondrogenesis marker genes including Sox9, Aggrecan and Col II was activated (Figure 2F). These data described above implied that these fibroblast-like cells had osteogenic, adipogenic and chondrogenic differentiation potential *in vitro*.

**FIGURE 3:**
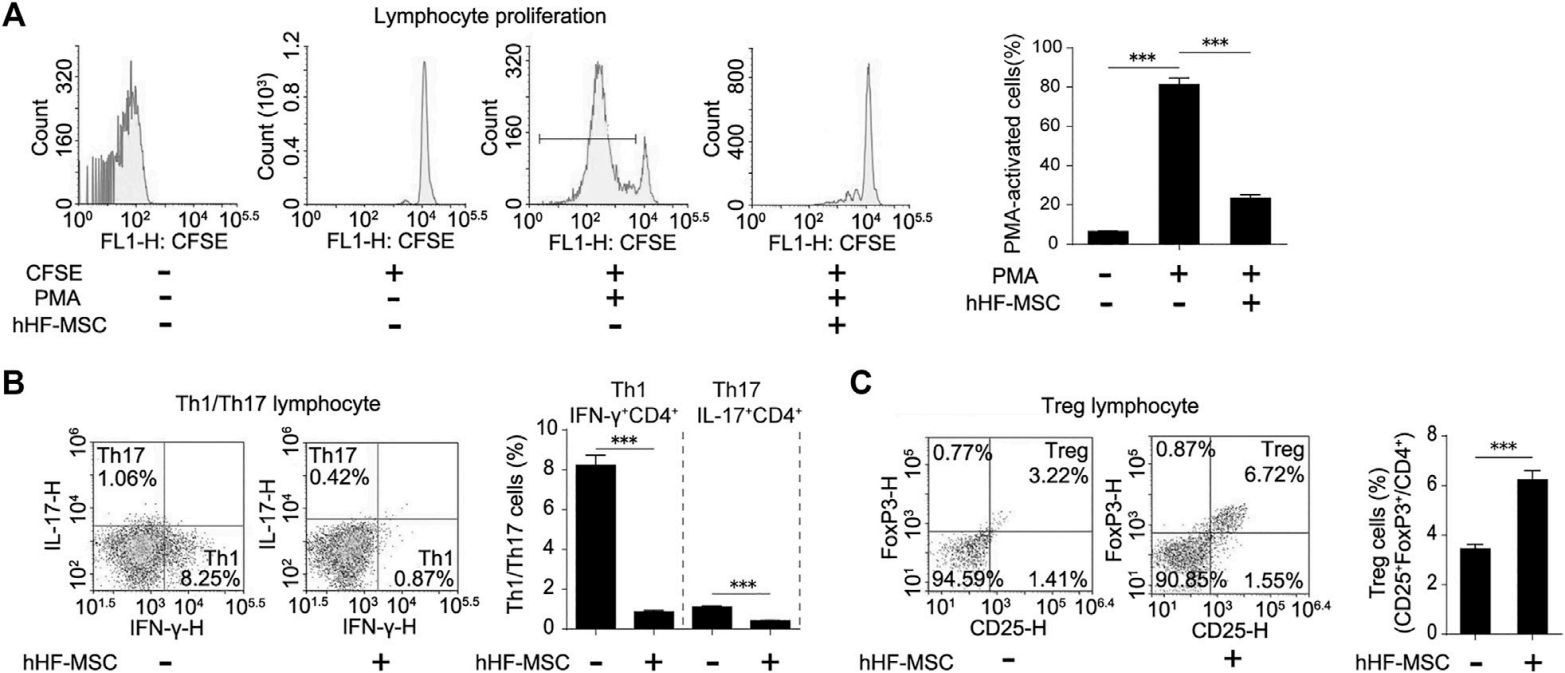
Immunomodulatory effects of hHF-MSCs. **(A)** Flow cytometry analysis and quantification on growth suppression of PBMCs in co-culture with hHF-MSCs for 3 days. **(B)** Flow cytometry analysis and quantification on differentiation of Th1 and Th17 in co-culture with hHF-MSCs for 3 days. **(C)** Flow cytometry analysis and quantification on differentiation of Treg in co-culture with hHF-MSCs for 3 days. All the data were obtained from three independent experiments. Data were shown as the means ± s.e.m. *: *p* < 0.05, **: *p* < 0.01, ***: *p* < 0.001, NS: not significant.

In order to evaluate the immunomodulatory effects of these fibroblast-like cells, they were co-cultured with normal human PMA-activated PBMCs for 3 days. Flow cytometry showed that the proliferation of PBMCs declined by 71% in co-cultured with these fibroblast-like cells from hair follicles, compared to the PMA-activated PBMCs alone (Figure 3A). To study the effect of these fibroblast-like cells on Th1/Th17 cell differentiation, purified CD4^+^ T cells were activated in Th1/Th17 activation conditions and co-cultured with these fibroblast-like cells for 3 days. Flow cytometry showed that these fibroblast-like cells markedly suppressed the expansion of IFN-γ secreting (Th1) cells and IL-17 secreting (Th17) cells (Figure 3B). And purified CD4^+^ T cells were activated in Treg activation conditions, then co-cultured with these fibroblast-like cells for 3 days. Result showed that these fibroblast-like cells markedly promoted the expansion of CD25 and FoxP3 producing (Treg) cells (Figure 3C).

Thus, due to surface markers of MSCs, tri-lineage differentiation potentials toward adipocytes, chondrocytes, osteoblasts, and immunomodulatory effects, these hair follicle-derived fibroblast-like cells were defined as hHF-MSCs.

### Intravenous Transplantation of hHF-MSCs Yields Better Recovery of Trabecular Bone Mass and Microstructure in Osteoporotic Mice

**FIGURE 4:**
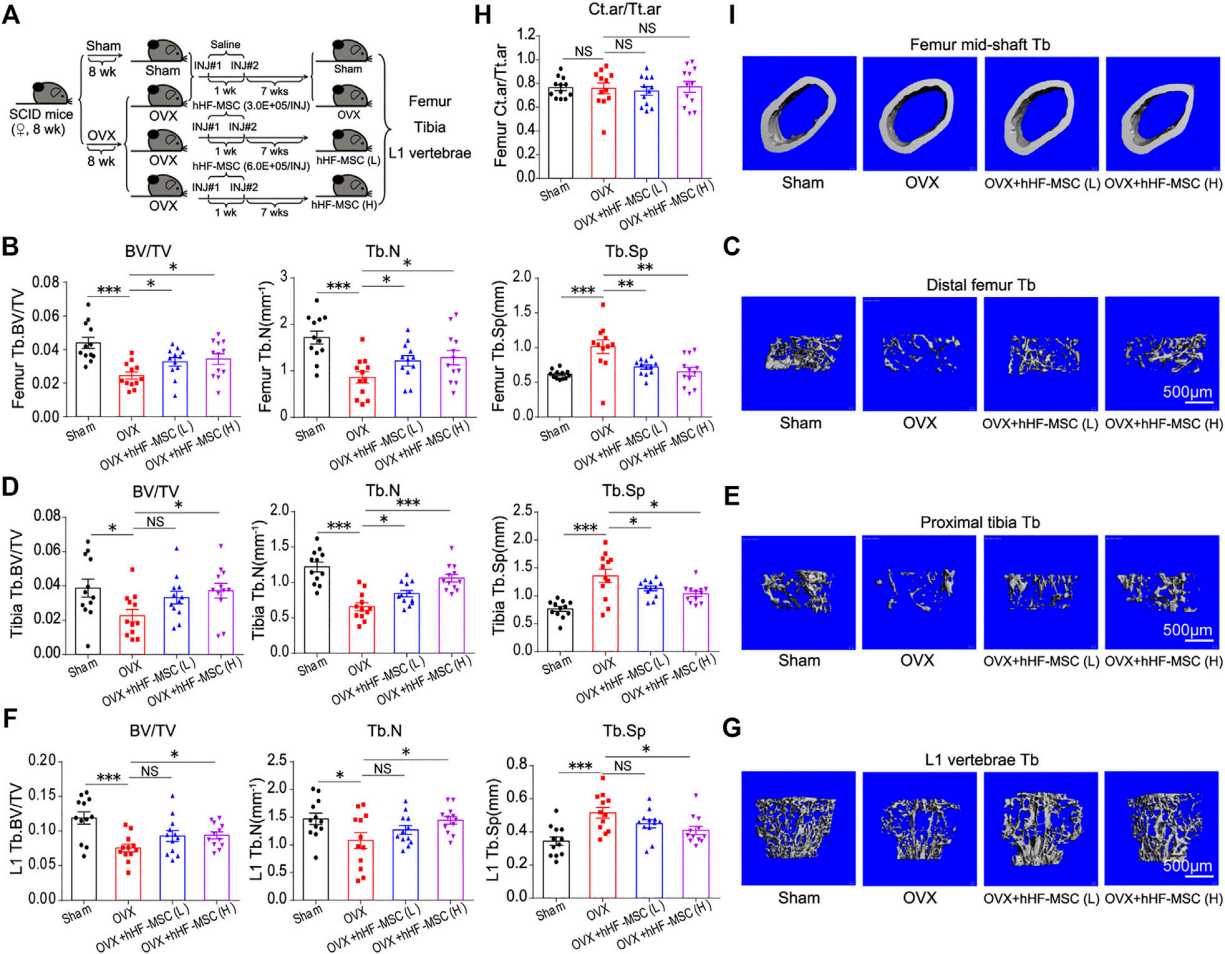
Intravenous transplantation of hHF-MSCs yields better recovery of trabecular bone mass and microstructure in OVX-induced osteoporotic mice. **(A)** Experimental grouping and timeline. OVX-induced osteoporosis in SCID mice was allowed to develop for 8 weeks before the first hHF-MSCs injection. The second hHF-MSCs injection was given at week 1 after the initial injection. Mice were sacrificed and samples were collected at week 7 after the second injection. **(B)** Bone volume fraction (BV/TV), trabecular bone number (Tb.N), and trabecular bone space (Tb.Sp) of distal femur of mice in response to hHF-MSCs transplantation determined by micro-CT (*n* = 12 for each group). **(C)** Representative 3D reconstruction images of distal femur trabecular bone. **(D)** BV/TV, Tb.N, and Tb.Sp of proximal tibia of mice in response to hHF-MSCs transplantation determined by micro-CT. (*n* = 12 for each group). **(E)** Representative 3D reconstruction images of proximal tibia trabecular bone. **(F)** BV/TV, Tb.N, and Tb.Sp of L1 vertebrae of mice in response to hHF-MSCs transplantation determined by micro-CT. (*n* = 12 for each group). **(G)** Representative 3D reconstruction images of L1 vertebrae trabecular bone. **(H)** Ct.ar/Tt.ar (the ratio of cortical bone area to total area) of mice in response to hHF-MSCs transplantation determined by micro-CT. (*n* = 12 for each group). **(I)** Representative 3D reconstruction images of cortical bone of femur mid-shaft. All the data were obtained from three independent experiments. Data were shown as the means ± s.e.m. *: *p* < 0.05, **: *p* < 0.01, ***: *p* < 0.001, NS: not significant.

In the current study, the effects of hHF-MSCs on the bone mass and microstructure *in vivo* were investigated through intravenous transplantation into osteoporotic SCID mice. OVX-induced and age-related bone loss model were employed to mimic the pathological conditions of type I and II osteoporosis in human respectively. Following that the bone loss was developed, 3.0 × 10^5^ (low dose) and 6.0 × 10^5^ (high dose) hHF-MSCs were injected through tail vein as the initial injection. The second hHF-MSCs injection with the same dose was given at week 1 after the initial injection. Mice were sacrificed and samples were collected at week 7 after the second injection. Femurs, tibias and L1 vertebrae were collected. Micro-CT was employed to assess the bone microstructure (Figure 4A and Supplementary Figure S1A).

In the distal femur, low- and high-dose hHF-MSCs transplantation led to an increase in BV/TV and Tb. N and a decrease in Tb.Sp in OVX-induced osteoporotic mice (Figures 4B,C). In the proximal tibia, high-dose hHF-MSCs transplantation led to an increase in BV/TV and Tb.N and a decrease in Tb.Sp in OVX-induced osteoporotic mice (Figures 4D,E). Although low-dose hHF-MSCs transplantation could enhance BV/TV of trabecular bone in the proximal tibia, this difference was not significant statistically. All the same, an increase in Tb.N and a decrease in Tb.Sp of the proximal tibia were observed in response to low-dose hHF-MSCs transplantation (Figures 4D,E). In the L1 vertebrae, low-dose hHF-MSCs transplantation failed to cause significant changes in BV/TV, Tb.N and Tb.Sp (Figures 4F,G). In contrast, high-dose hHF-MSCs transplantation led to an increase in BV/TV and Tb.N and a decrease in Tb.Sp in OVX-induced osteoporotic mice (Figures 4F,G). In the mid-shaft of femur, low- and high-dose hHF-MSCs transplantation failed to cause significant changes in Ct.ar/Tt.ar of cortical bone in OVX-induced osteoporotic mice (Figures 4H,I).

In the mouse model of age-related bone loss, low-dose hHF-MSCs transplantation failed to cause significant changes in BV/TV, Tb.N and Tb.Sp of distal femur (Supplementary Figures S1B,C), proximal tibia (Supplementary Figures S1D,E) and L1 vertebrae (Supplementary Figures S1F,G). In contrast, high-dose hHF-MSCs transplantation led to an increase in BV/TV and Tb.N and a decrease in Tb.Sp of distal femur (Supplementary Figures S1B,C), proximal tibia (Supplementary Figures S1D,E) and L1 vertebrae (Supplementary Figures S1F,G) in mice with age-related bone loss. In the mid-shaft of femur, low- and high-dose hHF-MSCs transplantation failed to cause significant changes in Ct.ar/Tt.ar of cortical bone (Supplementary Figures S1H,I).

### Increased Bone Formation and Decreased Bone Resorption in Response to Intravenous Transplantation of hHF-MSCs

**FIGURE 5:**
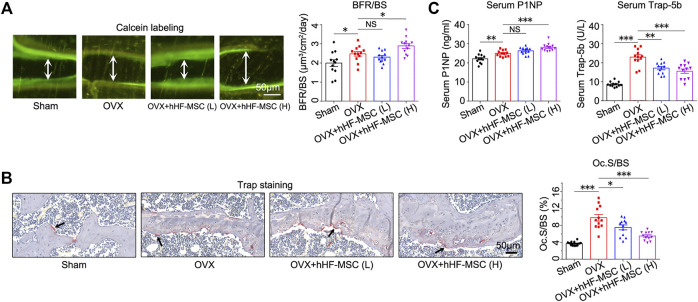
Increased bone formation and decreased bone resorption in OVX-induced osteoporotic mice with intravenous transplantation of hHF-MSCs. **(A)** Double calcein labeling images and bone formation rate (BFR) quantification of mice in response to hHF-MSCs transplantation. (*n* = 12 for each group). **(B)** Trap staining images and Oc.S/BS quantification of mice in response to hHF-MSCs transplantation. (*n* = 12 for each group). **(C)** Serum levels of bone turnover markers, P1NP and Trap-5b, in mice in response to hHF-MSCs transplantation determined by ELISA. All the data were obtained from three independent experiments. (*n* = 12 for each group). Data were shown as the means ± s.e.m. *: *p* < 0.05, **: *p* < 0.01, ***: *p* < 0.001, NS: not significant.

In addition to micro-CT scan, double calcein labeling assay and histomorphometric analysis of Trap^+^ osteoclasts were performed to characterize bone formation and bone resorption activity in OVX-induced osteoporotic mice with hHF-MSCs transplantation. In double calcein labeling assay, low-dose hHF-MSCs transplantation failed to cause significant changes in bone formation rate (BFR) (Figure 5A). In contrast, high-dose hHF-MSCs transplantation led to an increase in BFR in OVX-induced osteoporotic mice (Figure 5A), suggesting the enhancement of new bone formation in response to high-dose hHF-MSCs transplantation. In histomorphometric analysis of Trap^+^ osteoclasts, low- and high-dose hHF-MSCs transplantation led to an decrease in Oc.S/BS in OVX-induced osteoporotic mice (Figure 5B), suggesting the attenuation of bone resorption in response to hHF-MSCs transplantation.

Meanwhile, bone formation and resorption markers in peripheral blood serum were analyzed by Elisa to determine the bone turnover status in response to hHF-MSCs transplantation. Likewise, low-dose hHF-MSCs transplantation failed to cause significant changes in serum procollagen type 1 N-propeptide (P1NP) levels (Figure 5C). In contrast, high-dose hHF-MSCs transplantation led to an increase in serum P1NP levels in OVX-induced osteoporotic mice (Figure 5C). Low- and high-dose hHF-MSCs transplantation led to a decrease in serum tartrate-resistant acid phosphatase 5b (Trap-5b) in OVX-induced osteoporotic mice (Figure 5C).

Serum P1NP and Trap-5b levels were also investigated in the age-related bone loss of mice in response to hHF-MSCs transplantation. Low-dose hHF-MSCs transplantation failed to cause significant changes in serum P1NP and Trap-5b levels (Supplementary Figure S2). In contrast, high-dose hHF-MSCs transplantation led to an increase in serum P1NP levels and a decrease in serum Trap-5b levels in mice with age-related bone loss (Supplementary Figure S2).

### *In vivo* Distribution of Intravenous Transplanted hHF-MSCs in OVX-Induced Osteoporotic Mice

**FIGURE 6:**
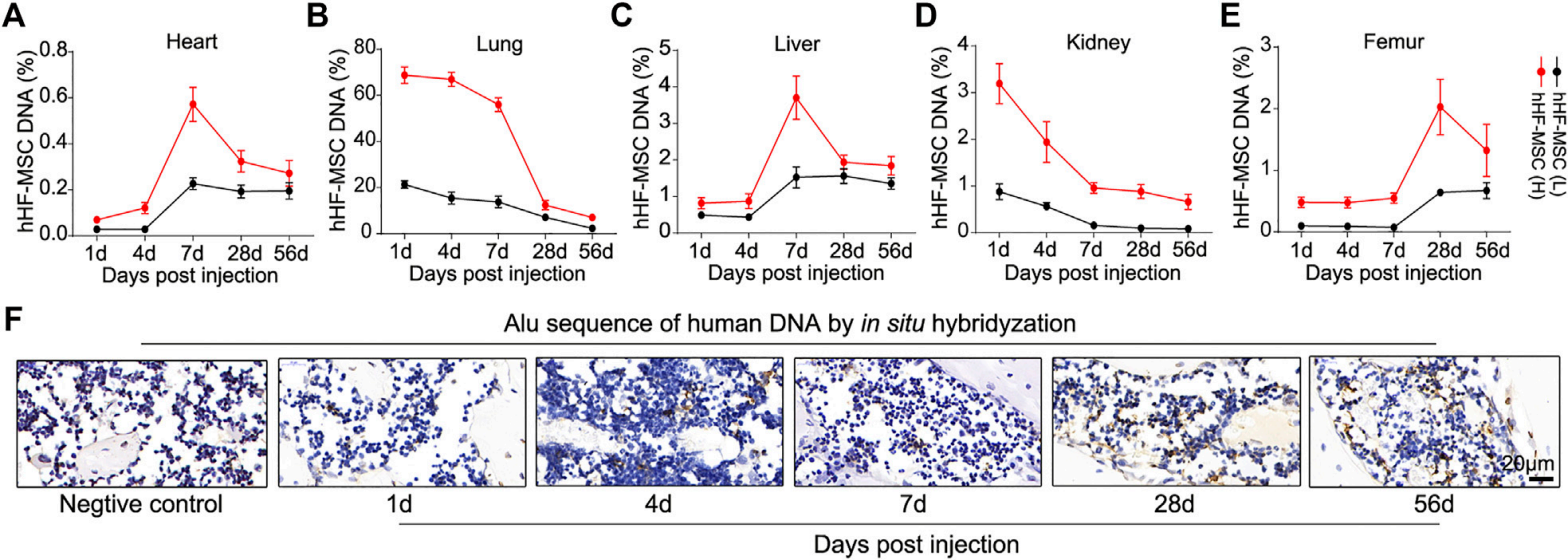
*In vivo* distribution of intravenous transplanted hHF-MSCs in OVX-induced osteoporotic mice. **(A–E)** The percentage of exogenous hHF-MSCs DNA in DNA of heart, lung, liver, kidney, and femur of mice at day 1, 4, 7, 28, and 56 after the second hHF-MSCs injection determined by qPCR using primers for Alu sequences. (*n* = 6 for each group). **(F)** *In situ* hybridization analysis against Alu sequences in the bone marrow of femur at day 1, 4, 7, 28, and 56 after the second high-dose hHF-MSCs injection. Hybridyzation reaction without probes was used as negative control. All the data were obtained from three independent experiments. Data were shown as the means ± s.e.m.

*In vivo* distribution of exogenous hHF-MSCs was investigated in OVX-induced osteoporotic mice. Genome DNA was isolated from heart, lung, liver, kidney, and femur of mice at day 1, 4, 7, 28, and 56 after the second hHF-MSCs injection. Alu sequences which are only present in human genome but absent in mouse genome were detected through qPCR. In heart, the percentage of exogenous hHF-MSCs increased from day 1–4 and reached its maximal levels at day 7 and thereupon fell progressively till day 56 (Figure 6A). In lung, the maximal levels in the percentage of exogenous hHF-MSCs were reached at day 1 and then declined progressively till day 56 post-injection (Figure 6B). The distribution pattern of exogenous hHF-MSCs in liver was comparable with heart (Figure 6C). The distribution pattern of exogenous hHF-MSCs in kidney was comparable with lung (Figure 6D). In femur, the percentage of exogenous hHF-MSCs remained in very low levels from day 1–7 and reached its maximal levels at day 28 and thereupon fell progressively till day 56 (Figure 6E). It should be noted that the *in vivo* distribution of exogenous hHF-MSCs was dose-dependent, that is, the distribution pattern of low-dose hHF-MSCs in these organs was comparable with high-dose hHF-MSCs but the levels of low-dose hHF-MSCs were lower than high-dose hHF-MSCs (Figures 6A–E).

Further, *in vivo* distribution of high-dose hHF-MSCs was investigated in the bone marrow of femur in OVX-induced osteoporotic mice through *in situ* hybridization against Alu sequences. There were only sporadic positive signals in the bone marrow of femur at day 1, 4, and 7 post-injection (Figure 6F). At day 28 and 56 post-injection, significant positive signals were observed in the bone marrow of femur (Figure 6F).

### Expression of Bone Metabolism Regulators in Serum and Bone Marrow in OVX-Induced Osteoporotic Mice With Intravenous Transplantation of hHF-MSCs

**FIGURE 7:**
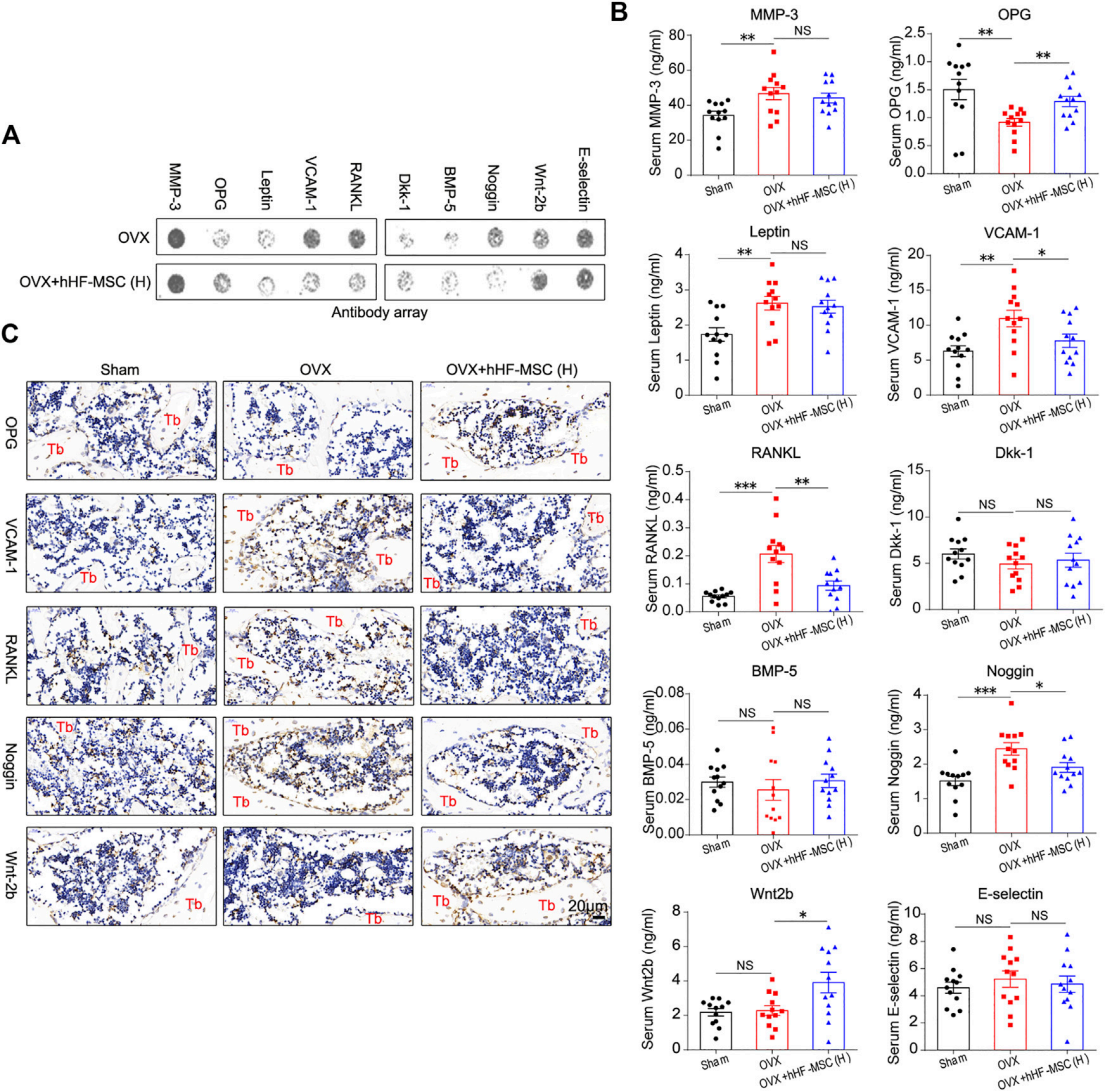
Expression of bone metabolism regulators in serum and bone marrow in OVX-induced osteoporotic mice with intravenous transplantation of hHF-MSCs. **(A)** Representative image of antibody array against bone metabolism regulators in peripheral blood serum from OVX-induced osteoporotic mice with high-dose hHF-MSCs injection. **(B)** The levels of bone metabolism regulators in peripheral blood serum from OVX-induced osteoporotic mice with high-dose hHF-MSCs injection determined by antibody array. (*n* = 12 for each group). **(C)** OPG, VCAM-1, RANKL, Noggin, and Wnt2b levels in femur bone marrow of OVX-induced osteoporotic mice in response to high-dose hHF-MSCs injection determined by immunohistochemistry. All the data were obtained from three independent experiments. Data were shown as the means ± s.e.m. *: *p* < 0.05, **: *p* < 0.01, ***: *p* < 0.001, NS: not significant.

Having observed increased bone formation, decreased bone resorption, and the enhancement of trabecular bone mass in osteoporotic mice in response to intravenous transplantation of hHF-MSCs, we made attempts to explore the underlying molecular mechanisms. Antibody array was employed to screen the secretion levels of bone metabolism regulators including matrix metalloproteinase-3 (MMP-3), osteoprotegerin (OPG), Leptin, Vascular cell adhesion molecule 1 (VCAM-1), Receptor Activator of Nuclear Factor-κ B Ligand (RANKL), Dkk-1, Bone Morphogenetic Protein-5 (BMP-5), Noggin, Wnt2b, and E-selectin in peripheral blood serum from OVX-induced osteoporotic mice in response to high-dose hHF-MSCs transplantation (Figures 7A,B). The serum levels of MMP-3, Leptin, Dkk-1, BMP-5, and E-selectin remained unchanged between with and without exogenous hHF-MSCs transplantation (Figures 7A,B). In contrast, the serum levels of OPG and Wnt2b were enhanced significantly in response to exogenous hHF-MSCs transplantation (Figures 7A,B). The serum levels of VCAM-1, RANKL, and Noggin were suppressed significantly in response to exogenous hHF-MSCs transplantation (Figures 7A,B).

Further, in addition to peripheral blood serum, the expression of OPG, VCAM-1, RANKL, Noggin, and Wnt2b in the bone marrow of femur was investigated through immunohistochemistry assay in OVX-induced osteoporotic mice with high-dose hHF-MSCs transplantation. The expression pattern of these factors in the bone marrow was comparable with serum, that is, OPG and Wnt2b were up-regulated and VCAM-1, RANKL, and Noggin were down-regulated in response to exogenous hHF-MSCs transplantation (Figure 7C).

## Discussion

Recently, MSCs derived from the dermal papilla or dermal sheath of the human hair follicle have received attention because of their accessibility and broad differentiation potential. Studies have reported that multipotent MSCs can be derived from the root tissue of hair follicles by directly plucking the human hair (Wang et al., 2013), and that these HF-MSCs share the common characteristics with other types of MSCs. Morphologically, HF-MSCs and BM-MSCs were very similar and almost indistinguishable. Like BM-MSCs, they expressed the cell-surface markers CD44, CD73, and CD90 (Zhang et al., 2013; Zhao et al., 2018), lower CD29 and CD105 and did not express the hematopoietic marker CD34. The main difference was the reduced expression of CD73 in BM-MSCs compared to hair follicle cells (Hoogduijn et al., 2006). Compared to BM-MSCs, HF-MSCs have the ability to differentiate into adipocytes, chondrocytes, osteoblasts and demonstrate high telomerase activity (Hoogduijn et al., 2006), which provide a novel alternative source with potential applications in stem cell therapy and tissue engineering.

It has been reported that BM-MSCs, AT-MSCs, dental pulp (DP)-derived MSCs, and umbilical cord (UC)-derived MSCs can efficiently increase trabecular number and raise BMD (An et al., 2013). However, unlike these MSCs, hair follicles are not limited by age, easily accessible and less invasive. People can store their hHF-MSCs when they are young, and use the stored stem cells to treat osteoporosis when they are old, which indicates its huge clinical value and commercial potential in cell therapy for osteoporosis in the future.

Bone homeostasis depends on the orchestrated bone remodeling process where osteoclastic bone resorption and osteoblastic bone formation are tightly coupled and maintained in dynamic equilibrium (Wang et al., 2012). At menopause, estrogen withdrawal accelerates bone remodeling with a net increase in bone resorption, which leads to bone loss and even osteoporosis (Lerner, 2006; Awasthi et al., 2018). In this study, we identified the therapeutic effect of hHF-MSCs on the trabecular bone loss through inhibiting bone resorption and promoting bone formation. Our findings also revealed that the transplantation of exogenous hHF-MSCs ameliorated the trabecular bone loss of osteoporotic mice mainly depending on the inhibition of bone resorption compared with the promotion of bone formation (Figure 4 and Supplementary Figure S1).

After we observed increased bone formation, decreased bone resorption, and the enhancement of trabecular bone mass in osteoporotic mice in response to intravenous transplantation of hHF-MSCs, we made attempts to explore the underlying molecular mechanisms preliminarily. In the current study, we found that several cytokines including OPG, RANKL, Noggin, VCAM-1, and Wnt2b might be involved in this process (Figure 7). OPG/RANK/RANKL and WNT are major signaling pathways that govern the bone remodeling process. OPG protects the skeleton from excessive bone resorption by acting as a soluble decoy receptor that can bind to RANKL (Min et al., 2000), and higher OPG is related to increased bone mass and reduced osteoclast numbers and activity (Simonet et al., 1997). Wnt2b is an important protein in the WNT signaling pathway, up-regulates the expression of Runx2 and Osterix in the process of osteogenesis (Jia et al., 2019). The up-regulation of Wnt2b expression can promote osteogenesis. Noggin is a BMP antagonist that can decrease BMD via the inhibition of bone formation (Devlin et al., 2003; Wu et al., 2003). Vascular cell adhesion molecule 1 (VCAM-1) is a cell adhesion molecule and it plays multiple roles in inflammation, cell differentiation and various immunological disorders, including rheumatoid arthritis, asthma, transplant rejection, and cancer (Kong et al., 2018). Accumulating studies have found that the cell-adhesion molecule VCAM-1 is necessary for osteoclastogenesis (Feuerbach and Feyen, 1997; Gkouveris et al., 2019). Reduced Noggin, VCAM-1 and RANKL levels is related to decreased bone resorption. But the more detailed mechanisms leading to these changes require further study.

Although we have replaced aging stem cells with exogenous stem cells, the causes of osteoporosis remain, the duration of the corrective effect of exogenous stem cells on osteoporosis, and whether it is possible to modify stem cells *in vitro* to prolong their efficacy *in vivo* are key scientific questions that are closely related to the application of stem cells and need to be studied in depth in the future. Moreover, aside from skeletal system, whether there are functional changes in other systems and organs of mice after the transplantation of exogenous hHF-MSCs are need to be determined. With aging of skin, dermis undergoes a decrease in activated fibroblast proportion which would result in a diminished capacity to regenerate the hair follicle (Chi et al., 2013; Chen et al., 2014), so it should have been considered to compare the difference in the efficacy of hHF-MSCs from donors of different ages on OVX-induced bone loss.

In summary, our findings demonstrated a novel therapeutic potential of human HF-MSCs transplantation in osteoporosis, that intravenous transplantation of hHF-MSCs not only promoted bone formation but also inhibited bone resorption, thus ameliorates bone loss in OVX and aging-induced osteoporotic mice. Our study may provide a promising therapeutic target in primary osteoporosis.

## Data Availability

The original contributions presented in the study are included in the article/Supplementary Material, further inquiries can be directed to the corresponding authors.
