# Supplementary material for: Intravenous Transplantation of Human Hair Follicle-Derived Mesenchymal Stem Cells Ameliorates Trabecular Bone Loss in Osteoporotic Mice
Source: Front Cell Dev Biol. 2022 Mar 10;10:814949. doi: 10.3389/fcell.2022.814949 (PMC8960386; doi:10.3389/fcell.2022.814949)
Supplement: Supplementary file 1 [file Table1.DOC]

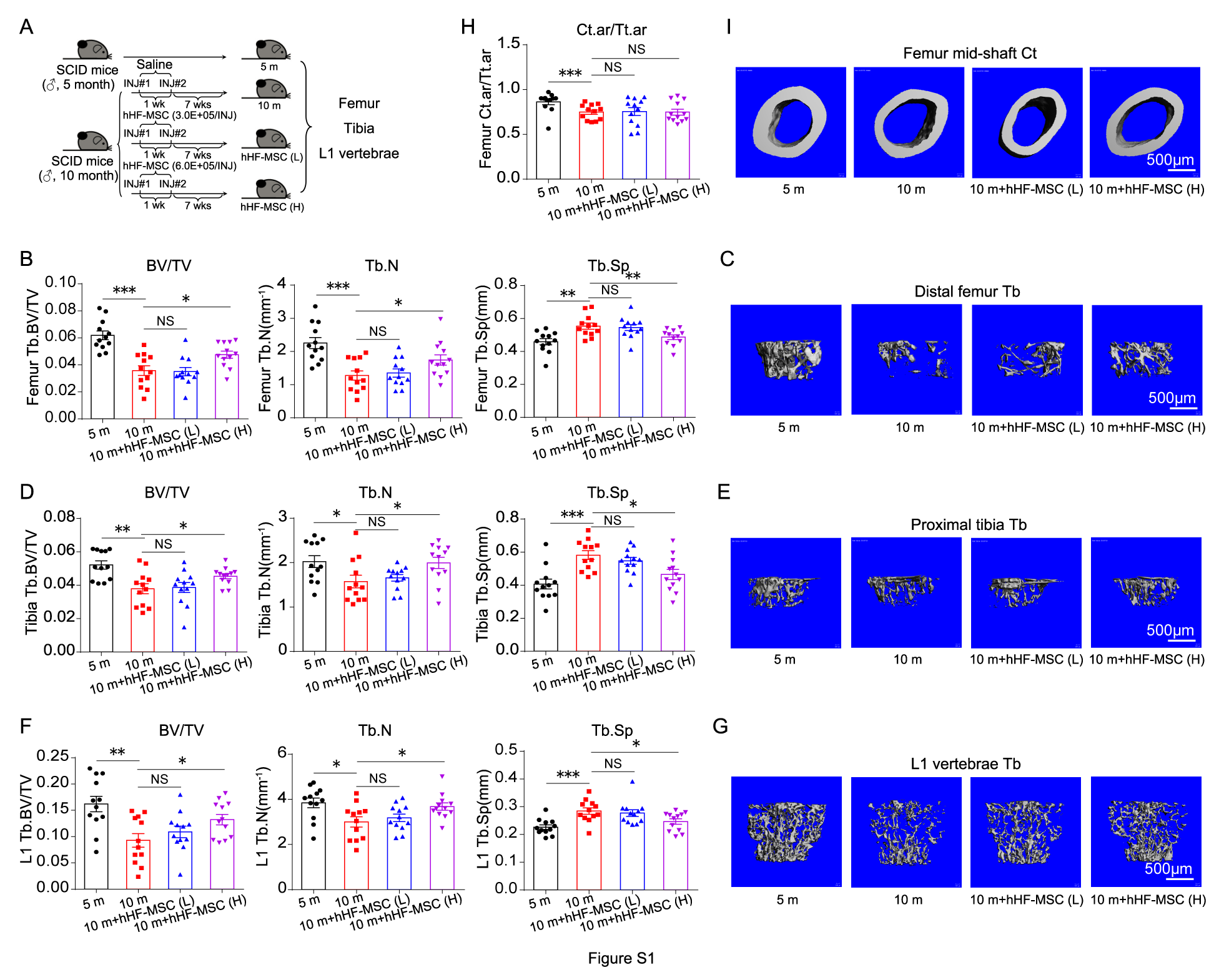


**Figure S1: Intravenous transplantation of hHF-MSCs yields better recovery of trabecular bone mass and microstructure in mice with age-related bone loss.**

**(A)** Experimental grouping and timeline. Age-related bone loss was allowed to develop in 10-month-old SCID mice compared with 5-month-old mice before the first hHF-MSCs injection. The second hHF-MSCs injection was given at week 1 after the initial injection. Mice were sacrificed and samples were collected at week 7 after the second injection. **(B)** Bone volume fraction (BV/TV), trabecular bone number (Tb.N), and trabecular bone space (Tb.Sp) of distal femur of mice in response to hHF-MSCs injection determined by micro-CT. (n=12 for each group). **(C)** Representative 3D reconstruction images of distal femur trabecular bone. **(D)** BV/TV, Tb.N, and Tb.Sp of proximal tibia of mice in response to hHF-MSCs injection determined by micro-CT. (n=12 for each group).**(E)** Representative 3D reconstruction images of proximal tibia trabecular bone. **(F)** BV/TV, Tb.N, and Tb.Sp of L1 vertebrae of mice in response to hHF-MSCs injection determined by micro-CT. (n=12 for each group). **(G)** Representative 3D reconstruction images of L1 vertebrae trabecular bone. **(H)** Ct.ar/Tt.ar (the ratio of cortical bone area to total area) of mice in response to hHF-MSCs injection determined by micro-CT. (n=12 for each group). **(I)** Representative 3D reconstruction images of cortical bone of femur mid-shaft. All the data were obtained from three independent experiments. Data were shown as the means ± s.e.m. *: p<0.05, **: p<0.01, ***: p<0.001, NS: not significant.


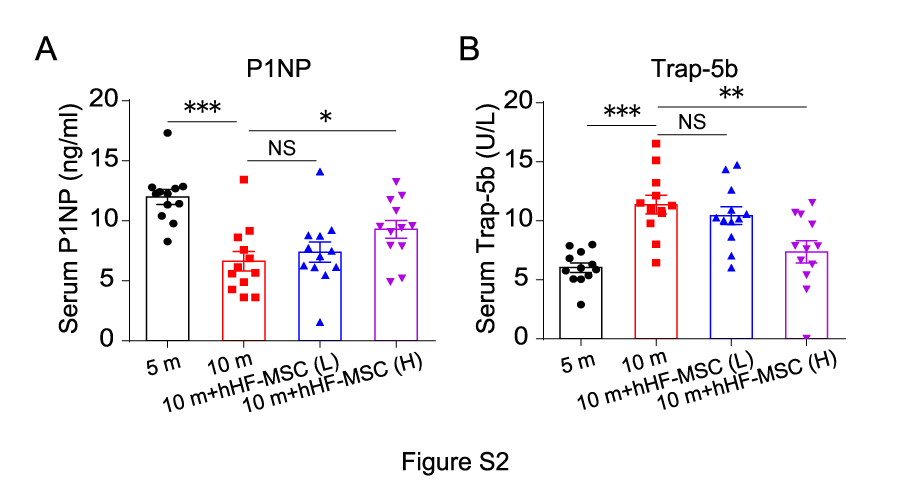


**Figure S2: Serum levels of bone turnover markers in mice with age-related bone loss in response to intravenous transplantation of hHF-MSCs.**

**(A)** P1NP (bone formation marker). (n=12 for each group). **(B)** Trap-5b (bone resorption marker). (n=12 for each group). m: month, L: low dose, H: high dose. All the data were obtained from three independent experiments. Data were shown as the means ± s.e.m. *: p<0.05, **: p<0.01, ***: p<0.001, NS: not significant.

**Table S1: Primer sequences used in RT-qPCR**

| **Gene** | **Primer sequences (5’-3’)** | **Product length (bp)** |
| --- | --- | --- |
| RUNX2 | GCGGTGCAAACTTTCTCCAG (F) | 114 |
|  | TGCTTGCAGCCTTAAATGACTC (R) |  |
| OPN | ATCTCCTAGCCCCACAGACCC (F) | 216 |
|  | CCGTGGGAAAATCAGTGACCA (R) |  |
| OCN | CACCGAGACACCATGAGAGC (F) | 132 |
|  | CTGCTTGGACACAAAGGCTGC (R) |  |
| PPARγ | TGATGTCTTGACTCATGGGTGT (F) | 232 |
|  | GTGTCAACCATGGTCATTTCTTGT (R) |  |
| aP2 | ATGGGGGTGTCCTGGTACAT (F) | 102 |
|  | CTTTCATGACGCATTCCACCA (R) |  |
| GLUT4 | ACTGGCCATTGTTATCGGCA (F) | 213 |
|  | GTCAGGCGCTTCAGACTCTT (R) |  |
| SOX9 | AAGGACCACCCGGATTACAA (F) | 105 |
|  | GTTGGGGGAGATGTGCGTC (R) |  |
| AGGRECAN | TGGTGATGATCTGGCACGAG (F) | 100 |
|  | CGTTTGTAGGTGGTGGCTGTG (R) |  |
| COL II | CATGAGGGCGCGGTAGAGA (F) | 243 |
|  | CCGGCTTCCACACATCCTTAT (R) |  |
| GAPDH | AATGGGCAGCCGTTAGGAAA (F) | 166 |
|  | GCCCAATACGACCAAATCAGAG (R) |  |
| HPRT | CCTGGCGTCGTGATTAGTGA (F) | 137 |
|  | CGAGCAAGACGTTCAGTCCT (R) |  |

F: forward, R: reverse.
